# Hematological and serum biochemical parameters of the captive long‐legged buzzard (*Buteo rufinus*) in Iran

**DOI:** 10.1002/vms3.916

**Published:** 2022-08-25

**Authors:** Hesamodin Kordestani, Bahman Abdi‐Hachesoo, Farnoosh Bakhshaei, Shirin Safaeian, Saeed Nazifi

**Affiliations:** ^1^ Department of Clinical Studies School of Veterinary Medicine Shiraz University Shiraz Iran

**Keywords:** haematology, biochemical parameters, long‐legged buzzard (*Buteo rufinus*), Iran

## Abstract

**Background:**

Although normal haematological and serum biochemical values for both pet and wild birds have been published, little information is available on the haematological and serum biochemical values in long‐legged buzzards (*Buteo rufinus*).

**Objectives:**

This is the first study that aimed to define reference values of haematological, biochemical parameters, and protein electrophoretic fractions of long‐legged buzzards in Iran.

**Methods:**

Blood samples were collected from 30 clinically healthy adult long‐legged buzzards of both sexes. Hematological, biochemical parameters, and protein electrophoretic fractions were measured. The mean and standard deviations were calculated.

**Results:**

Mean values for red blood cells, packed cell volume, haemoglobin, and white blood cells were 2.72 ± 0.60 ×10^6^/μl, 39.10 ± 3.70%, 13.45 ± 1.30 g/dl, and 3.92 ± 1.39 ×10^3^/μl, respectively. Mean values for biochemistry parameters were total protein 4.46 ± 1.27 g/dl, albumin 1.78 ± 0.55 g/dl, creatinine 0.54 ± 0.22 mg/dl, uric acid 7.81 ± 2.89 mg/dl, calcium 9.63 ± 2.22 mg/dl, phosphorus 4.31 ± 1.00 mg/dl, glucose 398.87 ± 96.90 mg/dl, blood urea nitrogen 10.46 ± 3.85 mg/dl, alkaline phosphatase 127.01 ± 1.46 IU/L, aspartate aminotransferase 262.22 ± 116.30 IU/L, and alanine aminotransferase 56.63 ± 27.85 IU/L. Mean values for serum protein fractions included pre‐albumin, albumin, α‐1 globulin, α‐2 globulin, β‐ globulin, and ϒ‐globulin was 0.20 ± 0.09, 2.35 ± 0.67, 0.28 ± 0.13, 0.32 ± 0.07, 0.62 ± 0.24, and 0.68 ± 0.53 g/dl, respectively.

**Conclusion:**

The reference data presented in this study can be used as health assessment values for veterinary laboratories and clinicians when developing release criteria for rehabilitated long‐legged buzzards.

## Graphical abstract

This is the first study that aimed to define reference values of haematological, biochemical parameters, and protein electrophoretic fractions of long‐legged buzzards in Iran. The reference data presented in this study can be used as health assessment values for veterinary laboratories and clinicians when developing release criteria for rehabilitated long‐legged buzzards.

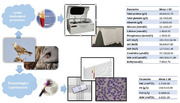

## 1 INTRODUCTION

The long‐legged buzzard (*Buteo rufinus*) is scientifically classified under Falconiformes order, Accipitridae family (Simpson, 1979). This medium‐sized raptor is distributed in the plains of North Africa, Southeast Europe, and from West to East Asia (Snow & Perrins, 1998). The long‐legged buzzard measures 50–65 cm in length, with a wingspan of 126–148 cm (Stevenson & Fanshawe, 2004). This species is an abundant raptor in Iran widely distributed in the north, west, and southwest regions of this country (Hosseini‐Zavarei et al., 2008; Shafaeipour, 2015). It feeds on small rodents, lizards, snakes, small birds, and large insects (Erdoǧan et al., 2012).

An assessment of the health status of wild bird populations indicates the quality of their habitat and various effects of the pathological conditions such as exposure to infection, contaminant intoxication, and malnutrition (Sparling et al., 1999). Many of these abnormal conditions could alter the normal haematological and serum biochemical parameters of wild birds. Several reports on viral (West Nile virus: Bakhshi et al., 2021), protozoan (Toxoplasma gondii: Namroodi et al., 2018), and helminths infections (Acanthocephala: Borhanikia et al., 2006) from the long‐legged buzzards in Iran emphasize the importance of monitoring programmes for these birds.

Blood sample analysis is a useful method to indicate animal health status and detect abnormalities and diseases (Newman et al., 1997). Haematology, serum biochemistry, and protein electrophoresis of avian species can provide reference values for predictive signals of recovery in rehabilitation centres as well as useful information for the chronicity and severity of bird diseases (Raidal, 2020). Serum protein electrophoresis (SPE) is considered a standard reference method for the determination of albumin and globulin (Eckersall, 2008). The interpretation of SPE patterns should be based on species‐specific reference intervals because there are differences in serum protein fractions among different avian species (Cray & Tatum, 1998).

Although normal haematological and serum biochemical values for some pet and wild birds have been published (García‐Montijano et al., 2002; Gelli et al., 2009; Hernandez et al., 1990; Heatley & Russell, 2020; Le Souëf et al., 2013; Newman et al., 1997; Nazifi et al., 2008; Samour et al., 2010; Samour, 2016), little information is available on the haematological and serum biochemical values in the long‐legged buzzard (*B. rufinus*). In this study, we provide baseline data for haematology and serum biochemical parameters and serum protein electrophoresis values in clinically healthy long‐legged buzzard (*B. rufinus*) in Iran.

## 2 MATERIALS AND METHODS

### 2.1 Birds

The present study was performed on 30 clinically healthy adult long‐legged buzzards. These long‐legged buzzards were housed at the Pardisan Wildlife Rehabilitation Center in Tehran.

The birds were checked by an expert veterinarian when they first arrived in the rehabilitation centre. If there was a sign or symptom indicating a potential injury, the birds were placed in recovery cages until they had fully recovered. Accordingly, all the sample collections in the present study were from apparently healthy birds which had already been treated for several weeks to months and were completely ready for successful release at the time of blood sampling. The birds were fed either chickens or rats, and management conditions such as cage situation, diet, and water supply were the same for all birds. These birds were previously found by members of the public and brought to the wildlife rehabilitation centre, and their exact age could not be specified. However, only adults were sampled based on a dark trailing edge to the wing as characteristic of adult long‐legged buzzards (Rodriguez et al., 2013). Long‐legged buzzards do not have sexual dimorphism, and sexing by DNA analysis was not performed for sex determination in this study.

At the time of blood sampling, an expert veterinarian declared each bird to be clinically normal. The clinically healthy birds at the time of release were determined according to the results of physical examination, weight loss assessment, flight evaluation, and their ability to capture live prey. No supplementary diagnostic procedures were performed. Sampling was done in late summer and early autumn. Blood sampling was done only once per individual bird. The birds had not received food for 8 h before sampling and were physically restrained for blood sampling in the early morning. The average time from admission into the rehabilitation centre until blood sampling was 1–2 months. Blood samples were taken after passing the health approval tests and before being released back into the wild.

### 2.2 Laboratory analysis

Blood samples were collected from the brachial vein using a 24‐gauge needle. Samples were divided into vacuumed sterile tubes with ethylene diamine tetraacetic acid as anticoagulant and serum collection tubes with clot activator for haematology and biochemistry analyses, respectively. Total erythrocyte count, total leucocyte count, haemoglobin concentration, and packed cell volume (PCV) values were assessed using avian haematological techniques (Thrall et al., 2012). To differentiate leucocyte counts, 100 leucocytes were counted on blood smears stained with Giemsa stain (Voigt & Swist, 2011).

Serum biochemical parameters including total protein (TP), albumin (Alb), creatinine, uric acid, calcium, phosphorus, glucose, blood urea nitrogen, alkaline phosphatase (ALP), aspartate aminotransferase (AST), and alanine aminotransferase were measured using standard methods and commercial kits (Pars Azmoon Co., Tehran, Iran) and a biochemical auto analyzer (Alpha Classic AT^++^, Sanjesh, Iran). Globulin was determined as the difference between serum TP and Alb. Cellulose acetate electrophoresis (20 min at 180 V using Elphor 5, Germany) was used to reveal serum proteins (pre‐albumin, albumin, and α1‐, α2‐, β‐, and γ‐globulins).

### 2.3 Data analysis

The mean and standard deviations (SD) were calculated. Statistical analysis was performed using SPSS software version 16(SPSS Inc., Chicago, USA).

## 3 RESULTS

**FIGURE 1:**
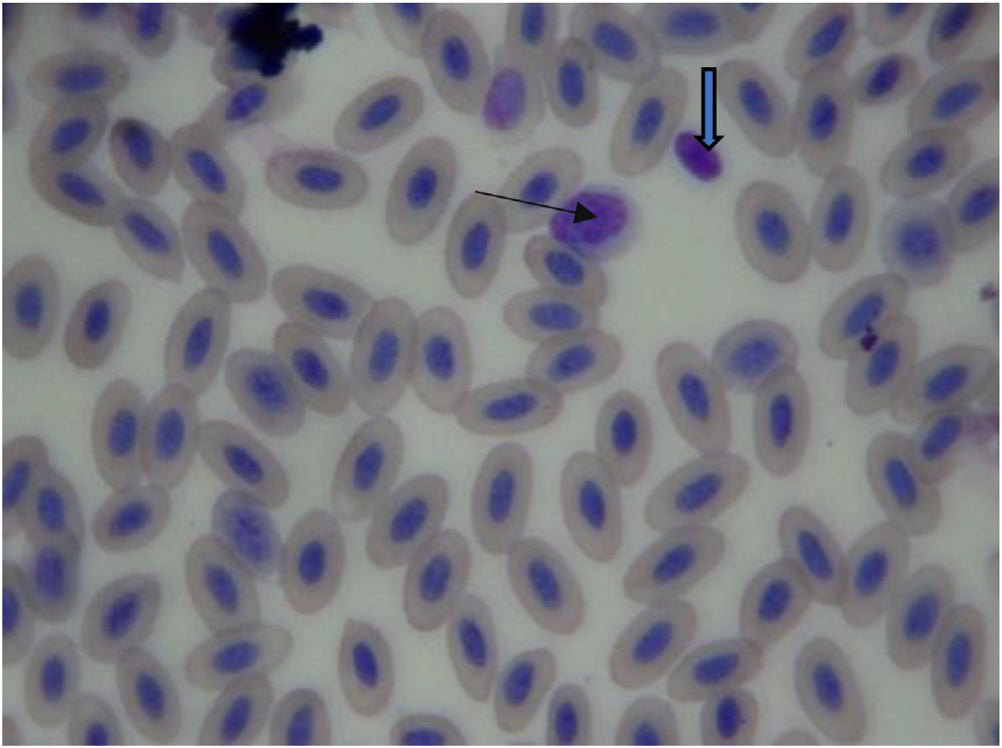
Blood smear of adult long‐legged buzzard (Giemsa stain) (thin arrow = lymphocyte; thick arrow = thrombocyte)

**FIGURE 2:**
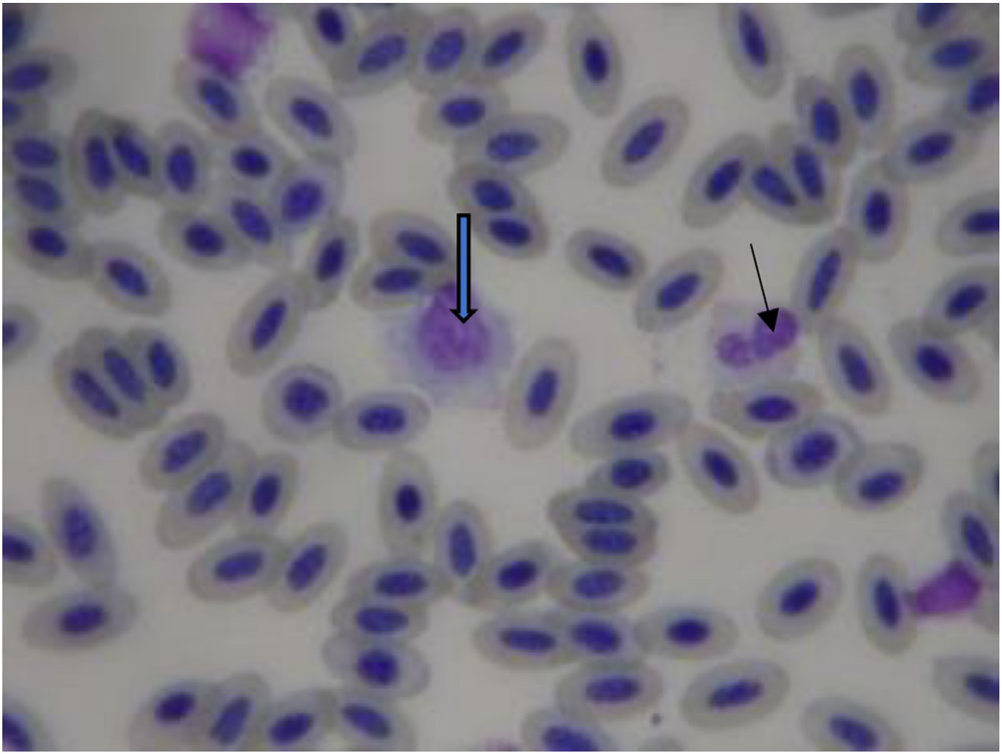
Blood smear of adult long‐legged buzzards (Giemsa stain) (thin arrow = heterophil; thick arrow = monocyte)

**FIGURE 3:**
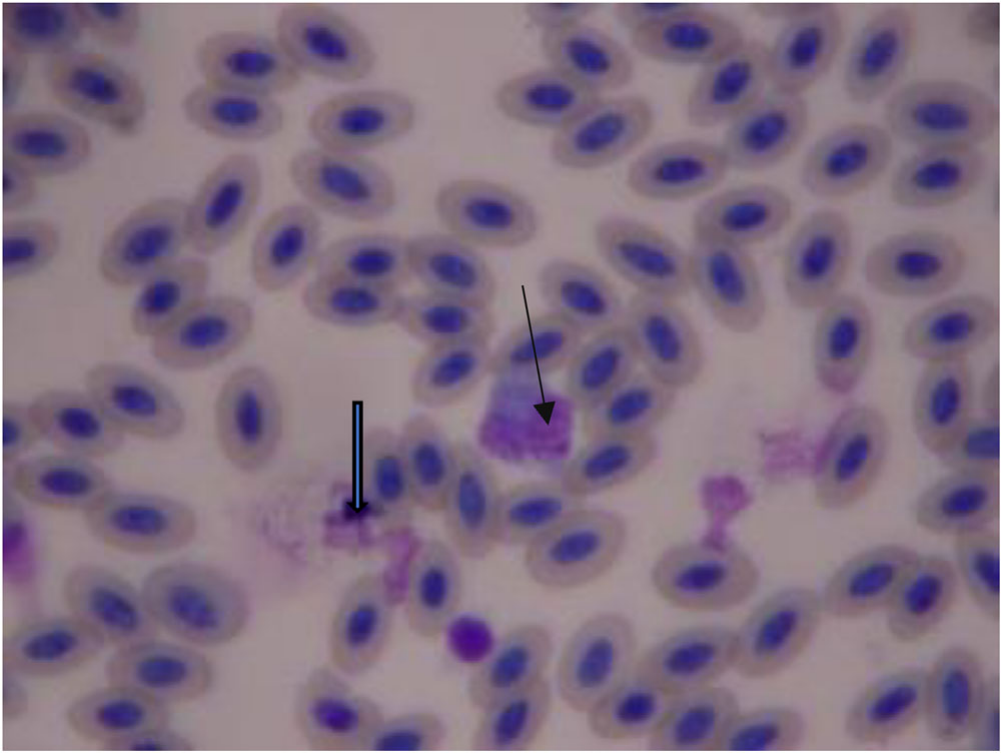
Blood smear of adult long‐legged buzzards (Giemsa stain) (thin arrow = monocyte; thick arrow = degenerated cell)

**FIGURE 4:**
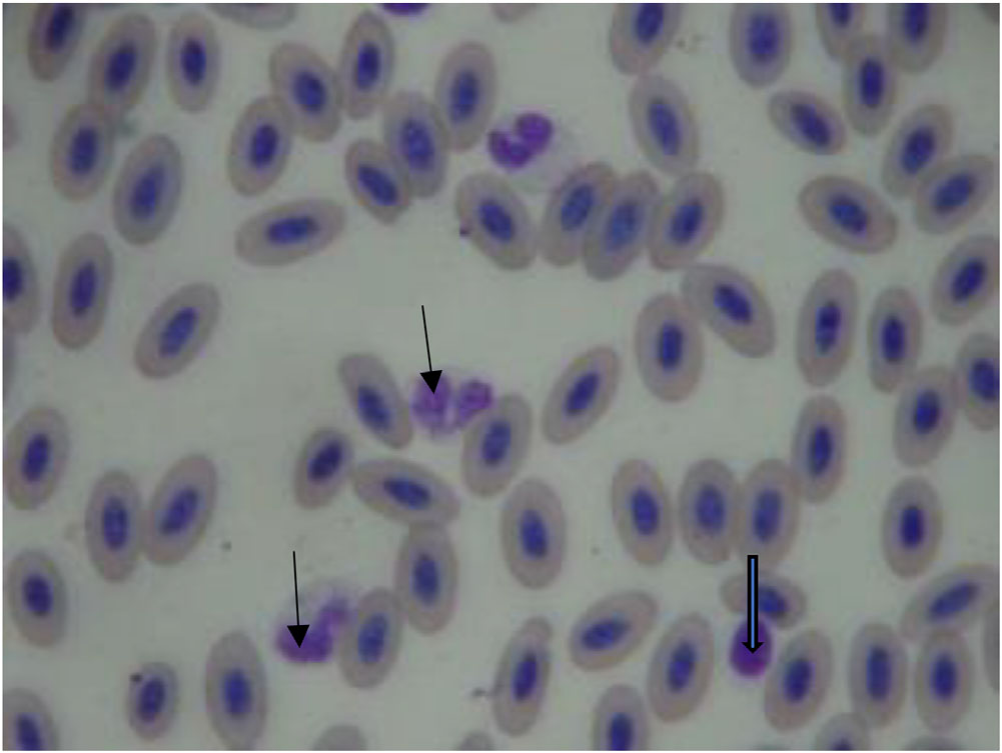
Blood smear of adult long‐legged buzzards (Giemsa stain) (thin arrow = heterophil; thick arrow = thrombocyte)

The mean ± SD of haematological and biochemical profiles and serum protein electrophoresis results for 30 long‐legged buzzards are listed in Tables 1, 2, and 3, respectively. The normal morphology of different blood cells in blood smears is shown in Figures 1, 2, 3, 4. The blood cells of the long‐legged buzzard were similar to their equivalents in other birds.

**TABLE 1:** Mean ± standard deviation (SD) of biochemical values for 30 long‐legged buzzards

Parameter	Mean ± SD	Differential
RBC (× 10^12^/L)	2.72 ± 0.60	
Hb (g/L)	134.50 ± 13.00	
PCV (L/L)	0.39 ± 0.03	
WBC (× 10^9^/L)	3.92 ± 1.39	
Heterophil	2.51 ± 0.90 (× 10^9^/L)	64.13 ± 2.13 (%)
Lymphocyte	0.93 ± 0.33 (× 10^9^/L)	23.73 ± 1.66 (%)
Monocyte	0.08 ± 0.05 (× 10^9^/L)	2.03 ± 0.92 (%)
Eosinophil	0.39 ± 0.17 (× 10^9^/L)	10.10 ± 2.76 (%)
Basophil	0.00 (× 10^9^/L)	0.00 (%)

Abbreviations: Hb, haemoglobin; PCV, packed cell volume; RBC, red blood cell; WBC, white blood cell.

**TABLE 2:** Mean ± standard deviation (SD) of haematological values for 30 long‐legged buzzards

Parameter	Mean ± SD
Total protein (g/L)	44.60 ± 12.70
Total globulin (g/L)	26.70 ± 9.90
Albumin (g/L)	17.80 ± 5.50
Glucose (mmol/L)	22.14 ± 5.37
Calcium (mmol/L)	2.40 ± 0.55
Phosphorous (mmol/L)	1.39 ± 0.32
AST (IU/L)	262.22 ± 116.30
ALT (IU/L)	56.63 ± 27.85
ALP (IU/L)	127.01 ± 1.46
Creatinine (μmol/L)	47.73 ± 19.44
Uric acid (μmol/L)	464.53 ± 171.89
BUN (mmol/L)	7.46 ± 2.74

Abbreviations: AST, aspartate aminotransferase; ALP, alkaline phosphatase; ALT, alanine aminotransferase; BUN, blood urea nitrogen.

**TABLE 3:** Mean ± standard deviation (SD) of serum protein electrophoresis results for 30 long‐legged buzzards

Parameter	Mean ± SD (g/L)	Mean ± SD (%)
Pre‐albumin	2.00 ± 0.90	4.56 ± 1.74
Albumin	23.50 ± 6.70	53.13 ± 9.14
α1‐Globulin	2.80 ± 1.30	6.42 ± 2.90
α2‐Globulin	3.20 ± 0.70	7.20 ± 2.57
β‐Globulin	6.20 ± 2.40	14.07 ± 4.13
ϒ‐Globulin	6.80 ± 5.30	14.60 ± 7.56

**FIGURE 5:**
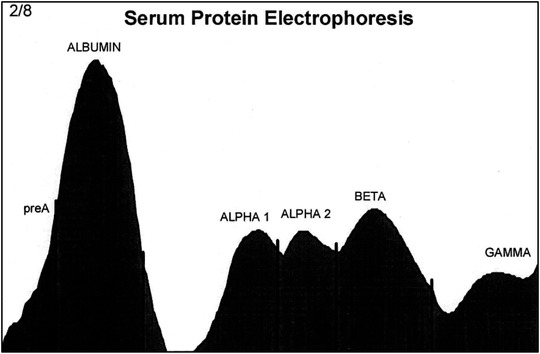
Electrophoretogram of cellulose acetate electrophoresis of serum proteins of adult long‐legged buzzards (pre‐albumin, albumin, and α1‐, α2‐, β‐, and γ globulins)

**FIGURE 6:**
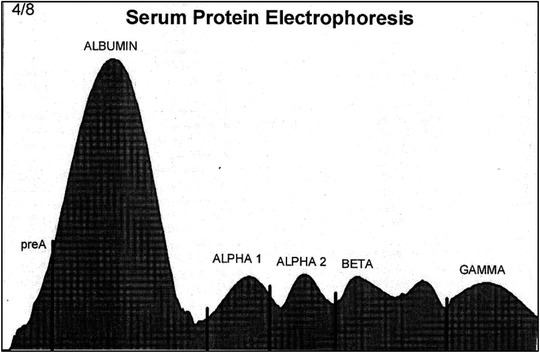
Electrophoretogram of cellulose acetate electrophoresis of serum proteins of adult long‐legged buzzards (pre‐albumin, albumin, and α1‐, α2‐, β1‐, and β2‐, and γ‐globulins)

Patterns for the distribution of proteins determined by serum protein electrophoresis are shown in Figures 5 and 6. Serum proteins were separated into pre‐albumin, albumin, and α1‐, α2‐, β‐, and γ‐globulins. The β‐globulin fraction contained β1‐ and β2‐globulins in two cases, but it had a single peak in 28 other long‐legged buzzards.

## 4 DISCUSSION

To our knowledge, this is the first report on haematology, biochemistry, and serum protein electrophoresis from long‐legged buzzards (*B. rufinus*) in Iran. There is a large number of published studies discussing the reference haematological and biochemical values of wild raptors (García‐Montijano et al., 2002; Gelli et al., 2009; Heatley & Russell, 2020; Hernandez et al., 1990; Le Souëf et al., 2013; Nazifi et al., 2008; Samour, 2016; Spagnolo et al., 2006). These data could be valuable clinically to evaluate the health of raptors in rehabilitation centres before release (Black et al., 2011). Some of these values could be considered as a beneficial predictive indicator in rehabilitation or veterinary centres (Heatley & Russell, 2020; Molina‐López et al., 2015).

In determining reference values ​​for analysis of haematological and biochemical parameters of birds and other exotic animals, the sample size should be between 20 and 40, and less than 20 samples are not suitable for reference values (Cray, 2015). Therefore, in this study, blood samples were taken from 30 apparently healthy long‐legged buzzards kept in Pardisan Wildlife Rehabilitation Center located in Tehran. The birds studied were all adults, and it was impossible to determine their sex. Therefore, the effect of age and sex on the measured parameters was not considered.

The number and type of leucocytes could vary among the raptor species. Except for some of the owl species, heterophil is the most common cell of other raptors (Joseph, 1999). Total leucocyte count in the long‐legged buzzard was lower than common buzzard (*Buteo buteo*) (Hernandez et al., 1990; Spagnolo et al., 2006), Spanish imperial eagle (*Aquila adalberti*) (García‐Montijano et al., 2002), red‐tailed hawk (*Buteo jamaicensis*), Cooper's hawk (*Accipiter cooperii*) (Black et al., 2011), and golden eagle (*Aquila chrysaetos*) (Nazifi et al., 2008). This could be due to the clinical health of the birds or subclinical or unknown diseases in other species. The heterophil was the most numerous leucocytes found in the blood of long‐legged buzzards, and lymphocytes were the next most abundant leukocytes. No basophil was observed in blood smears of the long‐legged buzzard. The percentage of leukocytes in the long‐legged buzzard was similar to the common buzzard (*B. buteo*) reported by Hernandez et al. (1990).

The hemogram of raptors could be altered by blood parasites, stress response, and infectious diseases (Joseph, 1999). Total red blood cell count and haemoglobin concentration in the long‐legged buzzard were similar to the results published in common buzzard (*B. buteo*) (Hernandez et al., 1990; Spagnolo et al., 2006) and Spanish imperial eagle (*A. adalberti*) (García‐Montijano et al., 2002) but were higher than those reported in golden eagle (*A. chrysaetos*) (Nazifi et al., 2008).

PCV has been shown as a good predictive indicator with clinical significance in the rehabilitation medicine of birds of prey (Molina‐López et al., 2015). Raptors with a PCV <35% are considered anaemic, and raptors with a PCV >45% are considered dehydrated (Joseph, 1999). Mean PCV in this study (39.1%) was similar to the formerly published results in common buzzard (*B. buteo*) (Hernandez et al., 1990; Spagnolo et al., 2006). In comparison, higher PCV percentages were reported for Spanish imperial eagle (*A. adalberti*) (García‐Montijano et al., 2002) and golden eagle (*A. chrysaetos*) (Nazifi et al., 2008).

Generally, the concentration of serum proteins in birds is less than in mammals (Kaneko et al., 2008). Decreased total protein values in raptors were seen in malnutrition, acute bleeding, or chronic gastrointestinal parasite infections, and high TP values may reveal dehydration and acute inflammations or infections (Joseph, 1999). According to previous investigations, the concentration of total proteins in common buzzard (*B. buteo*) is between 3–5 g/dl (Gelli et al., 2009; Hernandez et al., 1990; Spagnoloet al., 2006). The concentration of serum proteins in the long‐legged buzzard was 4.46±1.27 g/dl in agreement with common buzzard (*B. buteo*) and other species (Baumbusch et al., 2021; Black et al., 2011; García‐Montijano et al., 2002; Gelli et al., 2009; Heatley & Russell, 2020; Hernandez et al., 1990; Le Souëf et al., 2013; Nazifi et al., 2008; Samour et al., 2010; Samour, 2016; Spagnolo et al., 2006).

Reported serum calcium levels do not show wide variations among the raptors species (Joseph, 1999). However, the concentration of serum calcium in long‐legged buzzards was lower than that in common buzzard (*B. buteo*) (Gelli et al., 2009; Hernandez et al., 1990; Spagnolo et al., 2006). In most of bird species, the total plasma calcium concentration of less than 8.0 mg/dl is considered hypocalcaemia. Hypercalcemia may occur in dietary excesses of vitamin D or calcium, osteolytic bone tumours, oestrogen‐induced hypercalcemia, primary hyperparathyroidism, pseudo‐hyperparathyroidism, tertiary hyperparathyroidism, hyperalbuminemia, and dehydration. In contrast, hypocalcaemia has been reported with dietary calcium and vitamin D3 deficiency, dietary excesses of phosphorus, alkalosis, glucocorticoid therapy, and hypoalbuminemia (Harrison et al., 1994; Kaneko et al., 2008; Thrall et al., 2012). Serum phosphorus concentration of long‐legged buzzards was similar to common buzzard (*B. buteo*) in one report (Hernandez et al., 1990) and lower than common buzzard (*B. buteo*) in another report (Spagnolo et al., 2006). These differences were probably dietary phosphorus status.

Creatinine levels in long‐legged buzzards varied from other buzzard species (Gelli et al., 2009; Hernandez et al., 1990; Spagnolo et al., 2006). In this study, creatinine levels in long‐legged buzzards were in agreement with the findings of Spagnolo et al. (2006). Blood urea nitrogen in the long‐legged buzzard was higher than in common buzzard (*B. buteo*) (Spagnoloet al., 2006) and golden eagle (*A. chrysaetos*) (Nazifi et al., 2008). High plasma urea levels can be associated with conditions that reduce the flow of urine, such as dehydration or bilateral ureteral obstruction (Harrison et al., 1994). The birds in our study were in normal hydration status based on clinical examination, PCV, total protein, and albumin concentrations. Carnivorous birds have higher levels of uric acid in comparison with other birds, and slightly higher plasma urea levels in this study were probably due to the high protein diet. Greater levels than 20 mg/dl could be caused by kidney disease, shock, starvation, or during the training period of raptors in rehabilitation centres (Joseph, 1999). Serum uric acid concentration in the long‐legged buzzard was similar to common buzzard (*B. buteo*) (Gelli et al., 2009; Hernandez et al., 1990; Spagnolo et al., 2006).

Alanine aminotransferase and aspartate aminotransferase levels have a wide range in many species of birds (Kaneko et al., 2008; Thrall et al., 2012). High levels of aspartate aminotransferase may be seen with liver dysfunctions, soft‐tissue damages, bumble foot, and septicaemia in birds of prey. Adult raptors had a higher value than young birds (Joseph, 1999). Aspartate aminotransferase level in the long‐legged buzzard was similar to common buzzard (*B. buteo*) (Gelli et al., 2009; Hernandez et al., 1990; Spagnolo et al., 2006), Spanish imperial eagle (*A. adalberti*) (García‐Montijano et al., 2002), and golden eagle (*A. chrysaetos*) (Nazifi et al., 2008). AST levels in the long‐legged buzzard were higher than that of reported values for AST in short‐toed eagle (Baumbusch et al., 2021). Alanine aminotransferase level in the long‐legged buzzard was higher than other buzzard species (Gelli et al., 2009; Hernandez et al., 1990; Spagnolo et al., 2006), short‐toed eagle (*Circaetus gallicus*) (Baumbusch et al., 2021), and golden eagle (*A. chrysaetos*) (Nazifi et al., 2008).

ALP had a high SD in the long‐legged buzzard. The increase in osteoblastic activity, such as skeletal growth, nutritional secondary hyperparathyroidism, rickets, fracture repair, osteomyelitis, and impending ovulation, may cause increased ALP activity (Kaneko et al., 2008). Low ALP activities have been linked to dietary zinc deficiencies (Harrison et al., 1994).

Glucose level is higher in birds than mammals in a range of 200–500 mg/dl (Thrall et al., 2012). Hypoglycaemia is a possible problem of flight‐trained raptors commonly appeared with a history of inadequate food consumption and hard exercises during training periods (Joseph, 1999). In this study, serum glucose concentration was 398.87±96.9 mg/dl in the long‐legged buzzard.

Serum protein fractions of the long‐legged buzzard included pre‐albumin, albumin, α‐1 globulin, α‐2 globulin, β‐globulin, and ϒ‐globulin. In some samples (two samples), the beta fraction consisted of two bands, but in all 28 other samples, the beta band consisted of only one independent band. Therefore, in all samples, the beta band was considered a single band. The highest percentage of proteins in the long‐legged buzzard is related to albumin, and the lowest percentage is related to pre‐albumin. The mean serum albumin concentration for the long‐legged buzzard was higher than those established for common buzzard (*B. buteo*) (Gelli et al., 2009; Spagnolo et al., 2006) and Spanish imperial eagle (*A. adalberti*) (García‐Montijano et al., 2002). α‐1, α‐2, and β‐ globulin values for the long‐legged buzzard were lower than those reported for common buzzard (*B. buteo*), but ϒ‐globulin value for the long‐legged buzzard was higher than that reported for common buzzard (*B. buteo*) (Gelli et al., 2009; Spagnolo et al., 2006).

In general, avian haematological and biochemical values are subject to extensive variability resulting from different environment and management practices, which can affect physiological responses. The heterogeneity between the results of the present study and other studies could be due to different environments and management practices, seasonal changes, diurnal rhythm, diet, and gender. One limitation of this study was that the haematological and biochemical values of captive long‐legged buzzards were evaluated; however, the living environment and nutrition of these birds in nature could affect these parameters. Yet, the normal values published in this study can assist the veterinary laboratories and clinicians for the assessment of captive long‐legged buzzards for conservation programmes and health evaluation purposes for the determination of the release time of healthy birds.

## CONFLICT OF INTEREST

The authors declare no conflict of interest.

## AUTHOR CONTRIBUTIONS

*Conceptualization, data curation, investigation, methodology, project administration, resources, supervision, validation, visualization, and writing—review and editing*: Saeed Nazifi. *Conceptualization, data curation, methodology, project administration, resources, validation, visualization, and writing—review and editing*: Bahman Abdi‐Hachesoo. *Conceptualization, data curation, investigation, methodology, resources, validation, and visualization*: Hesamodin Kordestani. *Data curation, formal analysis, investigation, resources, software, validation, and writing—original draft*: Farnoosh Bakhshaei. *Data curation, formal analysis, investigation, resources, software, and writing—review and editing*: Shirin Safaeian. *Conceptualization, data curation, investigation, methodology, project administration, resources, supervision, validation, visualization, and writing—review and editing*.

## ETHICS STATEMENT

All animal experiments were approved by the State Committee on Animal Ethics, Shiraz University, Shiraz, Iran (IACUC no.: 4687/63). The recommendations of the European Council Directive (86/609/EC) of November 24, 1986, regarding the standards in the protection of animals used for experimental purposes were also followed.

### PEER REVIEW

I would not like my name to appear with my report on Publons https://publons.com/publon/10.1002/vms3.916

## Data Availability

The data that support the findings of this study are available on request from the corresponding author. The data are not publicly available due to privacy or ethical restrictions.
